# What Clonal Hematopoiesis Can Teach Us About MDS

**DOI:** 10.3389/fonc.2022.794021

**Published:** 2022-02-08

**Authors:** Irenaeus C. C. Chan, Brian J. Wiley, Kelly L. Bolton

**Affiliations:** Washington University School of Medicine, St. Louis, MO, United States

**Keywords:** clonal hematopoiesis (CH), myeloid neoplasm, genetic predisposition, environmental risk, myelodysplastic syndrome (MDS)

## Abstract

Clonal hematopoiesis (CH), defined as the clonal expansion of mutated hematopoietic stem and progenitor cells (HSPCs), is a common aging process. CH is a risk factor for the development of hematologic malignancies, most commonly myeloid neoplasms (MNs) including acute myeloid leukemia (AML), myelodysplastic syndrome (MDS), and myeloproliferative neoplasm (MPN). Recent work has elucidated how the development and cellular fitness of CH is shaped by aging, environmental exposures, and the germline (inherited) genetic background of an individual. This in turn has provided valuable insights into the pathogenesis of MNs including MDS. Here, in this review, we discuss the genetic origins of CH, the environmental stressors that influence CH, and the implications of CH on health outcomes including MDS. Since MNs have shared risk factors and underlying biology, most of our discussion regarding the implications of CH surrounds MN in general rather than focusing specifically on MDS. We conclude with future directions and areas of investigation including how intervention studies of CH might inform future therapeutic approaches to MN including MDS.

## Main

With every round of mitotic division, DNA damage and inefficient repair occur, resulting in the accumulation of somatic mutations in aging hematopoietic stem and progenitor cells (HSPCs) over time. The process of acquired mutations leading to clonal expansion of HSPCs is referred to as CH. The most common biochemical mutational event giving rise to CH is spontaneous deamination of methylated cytosine at CpG dinucleotides resulting in generation of thymine (1). CH is a normal process of aging (2–7). Indeed, the acquisition of aging-related mutations is not limited to stem cells of the hematopoietic system. Age-related somatic mutations are in fact pervasive across human tissues (8–12). Differences in the rate of somatic mutational acquisition across human tissues correlates with the rate of stem cell division and may in part explain variation in cancer risk among human tissues (10, 13). The patterns of aging-related somatic mutagenesis are conserved even across mammalian species with the mutation rate inversely correlated with species lifespan. This suggests that beyond risk of cancer, somatic mutation rate may be a determinant of lifespan (14). Mathematical reconstruction of HSPC lineage histories suggests that CH mutations may arise as early as childhood (even *in utero*) with some mutations undergoing a gradual expansion throughout life (11, 15).

Our ability to detect CH mutations and the observed prevalence of CH is dependent on the sensitivity of sequencing as determined by sequencing depth. For example, analysis of whole exome sequencing data sequenced at a depth of under 100-fold coverage generally reports age‐dependent single nucleotide variants (SNVs) or small insertions/deletions (indels) in approximately 6% of subjects over 65 years of age (2, 3, 14). In contrast, next-generation sequencing (NGS) technologies with advanced error-correction methods such as unique molecular indices, can accurately detect mutations below 0.5% variant allele fraction (VAF) when performed at a high depth (16). Using approaches such as these, the prevalence of CH mutations in the blood of adults over 50 appears closer to 100% (17). Sequencing technologies such as duplex consensus sequencing (9) and bottleneck sequencing (15) may enable the detection of individually mutated stem cells that have not undergone clonal expansion. The VAF used to define a clonally expanded stem cell is not well-defined and depends on assumptions regarding the number of stem cells actively contributing to white blood cell production. Studies tracking the clonal contributions of cells labelled directly *in vivo* have suggested that long-term homeostatic hematopoiesis is driven by many thousands of cells (18–20). However, recent work using single-cell-derived HSPCs suggests that the number of hematopoietic stem cells that are actively making white blood cells at any one time to be in the range of 50,000–200,000 (21).

The mutations driving CH confer a survival advantage over wild-type cells that results in the clonal expansion of the mutated cell. Thus, there is overlap in the genetic mutations driving CH and early drivers of hematologic cancer. CH driven by SNVs or indels are most commonly drivers of MNs. This includes epigenetic modifiers (*DNMT3A*, *TET2*, *ASXL1*, *IDH1*, *IDH2*), splicing factors (*SF3B1*, *SRSF2*, *U2AF1*), genes involved in the DNA damage response (*TP53*, *PPM1D*, *CHEK2*), and *JAK2.* Murine models have been useful in understanding the role that mutations in these genes play in conferring a fitness advantage. Mutations in the epigenetic regulators such as *DNMT3A* and *TET2* have been shown to confer an advantage by enhancing self-renewal of stem and progenitor cells and inhibiting their differentiation (22, 23). Mutations in other genes involved in the DNA damage response may enhance cell survival under specific cellular stressors (5, 24, 25).

Copy number events (amplifications, deletions, or copy neutral loss of heterozygosity) are also known to drive CH. Analysis of genome-wide single nucleotide polymorphism (SNP) array data initially suggested that large >2 Mb copy number events in autosomal chromosomes were observed rarely, in roughly 2% of individuals aged 70–75 (2). Computational tools that incorporate long-range phase information have since improved the sensitivity to detect copy number-driven CH almost 10-fold (26). Somatic loss of chromosome Y (LOY) in males is the most common copy number event driving CH, occurring in over 25% men over the age of 60 (6). Interestingly, many of the large copy number events driving CH overlap with those driving lymphoid malignancies (e.g., trisomy 12, deletion 13q, deletion 11q, among others). This likely reflects the importance of copy number events as early drivers in lymphoid malignancies (27). Other classes of mutational events such as medium-sized (>50 bp) copy number events, structural events and noncoding mutations have relevance in hematologic cancer and may also drive CH, yet are not well characterized. With the decreasing cost of high-depth whole genome sequencing and other technologies better positioned to detect these classes of events, the mutational spectrum of the events driving CH will likely become better defined.

### Germline Genetic Predisposition to Clonal Hematopoiesis

Germline (inherited) genetic factors appear important in determining both mutational acquisition and expansion of CH. Both common and rare germline CH susceptibility loci have been identified (19–21). Some genetic loci appear to be associated with CH driven by multiple driver genes (e.g., variants within the *TERT* and *CHEK2* loci are associated with CH driven by alterations in *DNMTA*, *JAK2*, and copy number events including LOY). Others, however, show heterogeneity in the strength of the association across driver genes. For example, germline *TCL1A* variants are associated with *DNMT3A*-mutant CH but not *JAK2*-mutant CH (28). While diverse, many loci converge upon key pathways regulating the cell cycle, DNA damage sensing, and the apoptotic process. Another theme is the high overlap between CH susceptibility genes and known cancer susceptibility genes (29). Other loci appear to be the objects of positive or negative clonal selection, i.e., CH events occurring in *cis* achieve a strong selective advantage by duplicating or removing inherited alleles. This was observed within the setting of healthy individuals in the UK Biobank where rare inherited variants were associated strongly with the acquisition of deletions or loss of heterozygosity in *cis (* 26). Somatic reversion or compensation for pathogenic germline alleles through CH has also been observed in the setting of inherited bone marrow failure syndromes (30, 31).

Studies of genetic susceptibility to LOY have shed light into the mechanism driving the long-established association between LOY and increased cancer susceptibility (32, 33), including MDS (34). Using data from the UK Biobank study, Thompson et al. identified 156 autosomal germline determinates of LOY (6). Genetic susceptibility to LOY was associated with risk of multiple types of cancer not only in men but also in women. Interestingly, LOY genetic susceptibility variants were also associated with age at natural menopause in women. Taken together, this suggests that LOY in men may represent a biomarker of genomic instability occurring in multiple tissue types thus supporting a “common-soil” hypothesis linking LOY to cancer risk. Future studies of genetic susceptibility to CH will provide further insights into the mechanisms linking CH to a diverse array of health outcomes.

Characterization of CH in individuals harboring MN-susceptibility genes has helped define the mechanisms driving genetic predisposition to MN. Two such examples include severe congenital neutropenia (SCN) and Shwachman-Diamond syndrome (SDS). SDS is a rare recessive disorder caused by bi-allelic mutations of *SBDS* that leads to impaired ribosome assembly, bone marrow failure, and MN early in life. CH due to mutations in *TP53* have been shown to occur much more frequently among children with SDS (48%–76%) compared with aged-matched controls (<1%) although the frequency of other common CH mutations (*DNMT3A*, *TET2*, *ASXL1*) was comparable. The most common molecular event demarcating the transition of *TP53* CH to MN in SDS was bi-allelic *TP53* inactivation and distinguished high from low-risk patients with CH. This suggests the presence of specific stressors in SDS that strongly select for HSPCs bearing *TP53* mutations and the importance of bi-allelic loss of *TP53* in determining malignant progression (35, 36). Individuals with SCN also have a unique molecular profile of CH with 40% of individuals having truncating mutations in the cytoplasmic domain of granulocyte colony-stimulating factor receptor (*CSF3R*) compared with 0% of age-matched controls (35). Treatment with granulocyte colony-stimulating factor (G-CSF) is the standard of care for SCN, and expression of the truncated G-CSF receptor confers a sustained increased signal in response to G-CSF (37). These observations suggest that high levels of endogenous G-CSF provide a powerful selective pressure on *CSF3R-*mutated HSPCs among individuals with SCN. Thus, studies of CH in patients with rare, pathogenic MN susceptibility syndromes provide a unique window into the convergence of genetic and environmental factors dictating CH selection and malignant progression.

### Environmental Influences on CH

We are in the infancy of understanding how environmental factors shape the risk of MDS. Indeed, beyond established associations between MDS and benzene exposure, cigarette smoking, and cytotoxic therapy, little is known regarding environmental risk factors. Problems with the diagnosis and classification of MDS have hampered the conduct of large-scale studies. However, recent work regarding the impact of environmental factors on CH have yielded important insights into how environmental factors might shape the development of MDS. Multiple observational studies have shown that CH is enriched among those exposed to cigarette smoke (2, 3, 5) and oncologic therapy (5, 22, 23). The strength of these associations varies by gene. For example, *ASXL1*-mutant CH is more strongly enriched among smokers compared with *DNMT3A*-mutant CH (5, 38), and CH mutations in genes belonging to the cell-cycle/DNA-damage response (DDR) pathway (39) are more common following exposure to cytotoxic therapy or radiation. Specific classes of cytotoxic therapy may confer a higher selective advantage on DDR-mutant HSPCS including platinum, radionuclide therapy, and topoisomerase I/II inhibitors. Chemotherapy-induced DNA damage in HSPCs triggers activation of apoptotic pathways resulting in cell death. HSPCs with mutations in genes regulating cell-cycle arrest and apoptosis including *TP53* and *PPM1D* carry a competitive advantage (38, 40) as HSPCs bearing these mutations result in decreased cell death following DNA damage (24, 25, 39). This suggests that different hematopoietic stressors promote the expansion of specific types of mutant clones. Indeed, this gene-specific selective pressure was confirmed previously by us through longitudinal blood sampling of solid tumor patients receiving cytotoxic therapy (5).

Conflicting epidemiologic findings exist regarding the association between chronic inflammation and risk of MN. Some studies have shown a modestly elevated risk of MN among those with a history of autoimmune diseases (41–43) while others report no significant association (44). Studies of the relationship between CH and inflammation may help address this question. Experimental data in murine model systems suggests that inflammation drives competitive advantage of CH clones. *TET2*-deficent and *TET2*-mutant HSCs show a strong proliferative advantage when exposed to exogenous TNF-α (45) and LPS-induced (46) inflammatory stress. Similarly, *DNMT3A-*mutant HSCs outcompete wild-type cells during infection. The frequency of CH has been reported to be elevated in individuals with some chronic inflammatory conditions [e.g., ulcerative colitis (47), HIV (48)] but not others (e.g., rheumatoid arthritis). Interpretation of these associations is limited by several factors. First, because these studies are cross-sectional, causality is difficult to determine (i.e., whether CH induces or is promoted by these conditions). Second, there are many possible confounders of these associations including related exposures such as medication effects. Serial sequencing of CH in longitudinal patient cohorts will provide further clarity regarding whether inflammatory conditions promote CH in humans.

### Consequences of CH

The vast majority of individuals with CH will never develop MN. However, in a subset, clonal selection and the acquisition of additional genetic aberrations will demarcate the transition to malignancy (5, 18). Individuals with multiple mutations, mutations with high clonal burden (high VAF), mutations in *TP53*, the spliceosome pathway, and *IDH1/2* are high-risk features (4–6). However, mutational features alone provide only modest predictive ability for risk of MN even when combined with blood count parameters and other clinical data (4, 5). Other factors likely play a major modifying role in the risk of developing MN. This could include clonal composition, epigenetic changes, or environmental factors among others.

Clonal cytopenia of undetermined significance (CCUS) is a condition in which individuals with CH also have unexplained low blood counts (cytopenia). Individuals with CCUS have a high probability of progression to hematologic malignancy, particularly MN, compared with individuals with cytopenias but no evidence of CH (5- and 10-year cumulative probability of progression: 82% vs. 9% and 95% vs. 9%, respectively) (49). Similar to CH, individuals with CCUS bearing only isolated *DNMT3A* mutations have a lower risk of progression to MN whereas individuals harboring *TET2*, *ASXL1*, *TP53*, and spliceosome mutations have higher risks of MN (50).

Characterization of hematopoietic stem cell population dynamics is critical in developing interventional approaches. CH driver mutations exert varying degrees of fitness advantage on HSPCs which partially explains variation in VAF between CH mutations (51, 52). In line with data that higher VAF CH is associated with an increased risk of MN, high-fitness variants (that confer faster CH growth) are associated with a higher risk of MN (51, 53). Longitudinal studies in healthy individuals have shown that while most clones display stable exponential growth over time, there is considerable variability with respect to accelerated and decelerated growth depending on the specific mutation and age of the individual (53). The clonal fitness and the risk of malignant progression may be shaped by not only driver mutations but also perhaps modified by co-occurring passenger mutations (14). Phylogenetic reconstruction suggests that in many cases, the CH mutations that eventually lead to MN may arise early in life, even *in utero (* 54, 55). Thus, further characterization of the lifelong clonal dynamics that demarcate high-risk features for progression will be critical to informing MN prevention strategies.

Beyond hematologic malignancies, CH is associated with an ever increasing list of diseases and health outcomes including cardiovascular disease (24–26), chronic obstructive pulmonary disease (27), infection risk including risk of severe COVID-19 (28, 29), autoimmune diseases (30), and HIV (31). We are in the early stages of understanding the mechanisms underlying this diverse list of disease associations. For example, multiple murine models and human data suggest that CH-induced changes in the hematopoietic stem cell inflammatory milieu may casually contribute to cardiovascular disease risk (26, 32, 33). However, a recent study suggests that HSC proliferation and resultant CH is elevated in mice with atherosclerosis, an observation supported in a small patient cohort (34). A combination of both functional data and longitudinal human studies will be critical in defining causality and the mechanisms driving these associations.

### Treatment Options and Prevention

While there are no established treatments for CH, surveillance and interventional strategies are an emerging area of research. Therapeutic intervention for patients with solid tumors with CH represents a possible avenue. Therapy-related MN (t-MN) is defined by the development of MN following exposure to cytotoxic chemotherapy and represents 10%–20% of MN (56). Among adult patients who develop t-MN following oncologic therapy, CH mutations (that develop into the dominant MN clone) can often be detected prior to therapy (5). Thus, assessment of CH prior to and during therapy could be used to identify patients at high risk of MN. For patients considering adjuvant therapy who have “high-risk” CH, consideration of dose reduction or avoidance of therapies known to be highly leukemogenic could be considered (5). This might be of particular relevance for individuals bearing CH mutations in *TP53* which are known to be promoted by oncologic therapy. However, this would need to be carefully weighed against the survival benefits of adjuvant therapy. Formal evaluation in specific clinical scenarios would be required prior to implementation.

Elimination of CH through use of a targeted therapy is an attractive goal. In patients with malignancies in which a dominant genetic alteration drives tumor, single agent-targeted therapies are highly effective. Examples include *c-KIT* mutations in gastrointestinal stromal tumors (GIST) and the *BCR-ABL* translocation in chronic myelogenous leukemia (CML). Thus, the use of targeted therapies in the setting of CH where the vast majority of patients harbor few mutations might be expected to show superior efficacy compared with MN. However, given the low rate of progression to MN, any adverse effects of treatment aimed at clone elimination must be weighed against the benefit. In this regard, focus on patients at the highest risk such as individuals with CCUS or patients with CH and hereditary predisposition syndromes to MN are of particular interest. Clinical trials utilizing *IDH* inhibitors in individuals with *IDH*-mutant CCUS are underway (NCT05030441, NCT05102370). While attractive, *IDH*-mutant CH is rare and far less common than CH-associated mutations in *DNMT3A*, *TET2*, or *ASXL1*. As additional targeted therapies are shown to be efficacious and safe in MN, more therapeutic options for CCUS and other high-risk forms of CH will emerge. However, most mutations driving CH exert their effects through loss of function of the corresponding gene rather than gain of function and thus will be more difficult to directly target.

A key safety outcome of therapeutic studies in CH, particularly targeted therapy, will be the long-term impact of suppressing specific CH clones with regard to the risk of emergence of other CH clones of malignant potential. The mechanisms of resistance to targeted therapies are diverse and yet to be completely characterized. Broad themes include the acquisition of secondary mutations in the targeted gene that influence drug binding and the emergence or selection of clones in alternative pathways. The extent to which long-term treatment with targeted therapies might suppress/eliminate specific CH mutations at the risk of selection of more pathogenic clones is not defined. Nevertheless, application of targeted therapies to the premalignant setting will further elucidate clonal dynamics and mechanisms of resistance.

## Conclusions

Characterization of the causes and consequences of clonal hematopoiesis will continue to provide insights into the pathogenesis of MN, including MDS. We are in the early stages of developing approaches for surveillance and intervention strategies for individuals with high-risk forms of CH. The successes (and failures) of these initial studies will provide additional avenues for optimizing MDS therapy.

## Author Contributions

IC, BW, and KB jointly wrote the manuscript. All authors listed have made a substantial, direct, and intellectual contribution to the work and approved it for publication.

## Funding

KB is supported by grants from the Damon Runyon Cancer Research Foundation, the American Society of Hematology, the EvansMDS fund, and the National Cancer Institute (5K08CA241318-02).

## Conflict of Interest

KB receives research funding from Bristol Myers Squibb and Servier.

The remaining authors declare that the research was conducted in the absence of any commercial or financial relationships that could be construed as a potential conflict of interest.

## Publisher’s Note

All claims expressed in this article are solely those of the authors and do not necessarily represent those of their affiliated organizations, or those of the publisher, the editors and the reviewers. Any product that may be evaluated in this article, or claim that may be made by its manufacturer, is not guaranteed or endorsed by the publisher.
